# Exploring the feasibility of integration of surveillance for intussusception into the routine monitoring of adverse events following immunization by countries of the WHO African Region for Africa

**DOI:** 10.11604/pamj.2022.41.157.26019

**Published:** 2022-02-22

**Authors:** Evans Mwila Mpabalwani, Jason Mathiu Mwenda, Bartholomew Dicky Akanmori

**Affiliations:** 1 University of Zambia, School of Medicine, Department of Paediatrics and Child Health, Lusaka, Zambia,; 2 WHO Regional Office for Africa (WHO/AFRO), Brazzaville, Republic of Congo

**Keywords:** Rotavirus vaccines, Intussusception, integration, WHO/AFRO countries

## Abstract

Surveillance for intussusception (IS) post-rotavirus vaccine introduction in World Health Organization Africa Region (WHO/AFRO) has been restricted mainly to the large referral teaching hospitals. The choice of these facilities for surveillance was made to utilize the abundant expertise of specialists in paediatrics and surgery in these hospitals who can diagnose and manage such patients with IS. The surveillance has been well coordinated by the African Intussusception Surveillance Network established in 2012. This network has supported surveillance across the African region and has accumulated a huge database of IS cases in children < 1 year with findings that have demonstrated safety of the monovalent rotavirus vaccine, Rotarix (GlaxoSmithKline). However, safety data on the pentavalent and RotaTeq (Merck Vaccine) is not yet available from the African region. Although, this network has provided much needed data, there is an inherent bias in monitoring and reporting of IS cases in only large tertiary hospitals. This time limited special project does not capture suspected intussusception cases with no access to hospital facilities used for monitoring IS. Additionally, the design requires extensive resources to support collection of high-quality data for monitoring IS, which is unsustainable. For these reasons suitable linkages between IS monitoring and routine Adverse Event Following Immunization (AEFI) should be established for continuity of monitoring of this condition. We propose alignment of the two systems that offers opportunity for high profile recognition and to enhance a sustainable system for diagnosis, treatment and continuous assessment of intussusception occurring in infancy.

## Commentary

### Introduction

Vaccines have a profound public health impact by preventing many priority diseases globally. Surveillance systems which adequately monitor Adverse Events Following Immunization (AEFI) contribute significantly to improving public confidence in vaccination programmes and help to minimize hesitancy and improve access to life saving vaccines. The lifecycle approach to vaccine regulation ensures that from research and development to licensure and post-licensure marketing the quality, efficacy and safety of vaccines are monitored [1-5]. Post-licensure monitoring of vaccines and phase IV studies are crucial for new vaccines particularly if they have been associated with any signals of safety which require verification through use in routine public health immunization service where millions of doses are administered compared to the smaller numbers given in phase III clinical trials [1-4].

Vaccine safety monitoring relies mainly on passive and active surveillance for AEFIs which are collected and entered into a national or global database for analysis and response. In the countries of the WHO African region, immunization programmes collect AEFI reports which are entered into Vigibase, maintained by the WHO Programme for International Drug Monitoring, by National Medicines Regulatory Authorities (NMRAs). Where there is active surveillance, as in the case of the three countries involved in the Malaria Vaccine Implementation Programme, NMRAs also receive AEFI reports from the sponsor of the phase IV study. The NMRAs, manufacturers, National Immunization Programmes (NIP), Disease Surveillance, National Pharmacovigilance Centres, hospitals and facilities, clinicians and the general public all have a role to play in monitoring health products in circulation and all have different roles. The general public, in the process of detection, recording, investigating and submitting data to the global pharmacovigilance centre is always central and they need to be informed of the outcome of the investigation [2, 3].

Countries are required to have in place systems for reporting all AEFI through the WHO/UNICEF Joint Reporting Form (JRF). Whenever a serious AEFI occurs (AEFI events requiring hospitalization, prolonged hospitalization or associated with a disability or death), they should be investigated and a causality assessment carried out by the national AEFI Committee and member countries have a specific AEFI investigation form recommended by WHO [1-4]. The outcomes should be communicated to the public the consumers of the products, the vaccine. The countries of the region are at different levels of implementation of their national pharmacovigilance and vaccine safety plans, developed in 2014 with the support of WHO [1, 2].

Intussusception (IS) is the invagination of the proximal loop of the intestine into the distal part leading to intestinal obstruction and is a surgical emergency [6, 7]. The obstruction has to be relieved early as any further delay leads to intestinal necrosis of the bowel segment with subsequent septicaemia, perforation, peritonitis and the outcome can be grave. This cascade of events is a function of time [8, 9]. In 1998, the first-generation rotavirus vaccine (RVV), Rotashield (Wyeth Lederle Vaccines Philadelphia) was introduced into the national immunization programme of the USA but was withdrawn in July 1999 from the market following its association with a small increase in cases of IS [5, 10] an AEFI, reported in the recipients of the vaccine. The second-generation, rotavirus vaccines, Rotarix (GlaxoSmithKline) and RotaTeq (Merck Vaccine), were found to be safe during clinical trials but low risk association with IS in recipients of these vaccines reported in a few developed countries when they were rolled out in the routine public health programmes [6].

The African Intussusception Surveillance Network supported by WHO and CDC Atlanta was established in 2012 with the main objective of monitoring IS following the introduction of RVV in the NIPs [6]. This initiative recommends use of a standardized protocol and questionnaire in all the countries for easy comparison and aggregation of data among participating countries [6]. The network has supported 12 countries to monitor cases of IS in children in their large sentinel site hospital and report data monthly to WHO. These countries included Ethiopia, Ghana, Kenya, Malawi, Tanzania, Zambia, Zimbabwe commonly referred to as Rotarix group and Cote d´Ivoire, The Gambia, Mali, Burkina Faso, Rwanda were categorized as RotaTeq group. The results obtained from this surveillance network sites have further confirmed the safety of Rotarix vaccine in use and provided confidence in the use of RVV in the NIPs in the region [6]. Evaluation is ongoing to complete the analysis of the data collected from countries using the RotaTeq vaccine. Overall, the African Intussusception Surveillance Network has accomplished its mandate and now the second phase is to incorporate IS surveillance into national AEFI monitoring systems. The data on IS will be collected routinely from selected health facilities assessed as part of IS network and those found to have adequate infrastructure will be required to conduct causality assessment with RVV to establish any association.

### Proposed design for integration of IS surveillance into the existing national AEFI monitoring systems

In this commentary, we propose a mechanism for integration which could involve reporting of confirmed cases of IS detected by all countries using the JRF. The confirmation of IS, a serious adverse event, country teams should be made by experienced consultant pediatricians and surgeons trained within IS network to confirm the diagnosis of IS using the standard Brighton case definition, conduct causality assessment where possible to establish if there is any association between cases of IS and rotavirus vaccination. A mechanism has to be established between hospital sentinel sites, EPI/NIP and National Regulatory Authority (NRA) on standard data collection, data flow, establishment of functional AEFI committees that includes experienced paediatricians, paediatric surgeons or general surgeons in target countries, periodic reporting to WHO of verified cases and vaccine history, networking of target countries and annual meeting to review data and exchange experiences. The roles and responsibilities of each of the stakeholders should be clearly defined and a platform for collaboration established. Durable intersectoral collaboration will be required as this will ensure all captured data on IS is in one repository. The proposed roles and responsibilities are outlined.

1) *The Expanded Programme on Immunization (EPI) or National Immunization Programme NIP:* the EPI is the anchor of immunization programmes in all countries and their activities are found at all health facility levels. This system will trigger alerts of IS reported by paediatricians/surgeons in hospitals, provide vaccination history and assist in any investigations by AEFI committee using the standard Brighton collaboration case definition. Record any cases of AEFIs and report to the NRA and Pharmcovigilance Committees (PVC). EPI will ensure sensitization of other key health personnel involved in the delivery and administration of vaccination programmes in their respective countries with experienced paediatricians/surgeons on standard case definition and diagnosis as members of the AEFI committee.

2) *National Regulatory Authority (NRA):* in some countries these are called National Medicines Regulatory Authority or National Pharmaceutical Regulatory Authority which is an agent of the Ministry of Health (MOH). Amongst other functions, they will review IS data periodically and share with MOH and WHO. More importantly they will meet with focal persons at hospitals and sentinel sites to review accuracy and adherence to standard case definition reporting.

3) *Sentinel site hospitals:* paediatricians and surgeons at all hospitals (sentinel sites) will continue to assess all cases of IS using standard case definition, collect and report data on confirmed IS cases. They will share data with the EPI secretariat who will then share the same with NRA and PVC. The data of confirmed IS cases will also be entered in the JRF and integrated into surveillance data base. At least one person from one large and three from small sentinel sites will be inducted into the national AEFI committee.

4) *Pharmacovigilance Committee (PVC):* this committee will determine the causality of the vaccine to AEFI and their role include interpretation of data of IS as part of AEFI. They would also share data with NRA and ensure that data is uploaded into the UMC vigiflow [11] systems.

5) *WHO/AFRO:* the EPI/MOH shares data with the WHO country office who are involved in monitoring, implementation and evaluation of IS surveillance in-country. They provide technical support and convene meetings of all stakeholders. Finally, WHO/AFRO would review regional data and provide feedback.

### Current Data flow chart for monitoring AEFIs in WHO/AFRO countries

**Figure 1:**
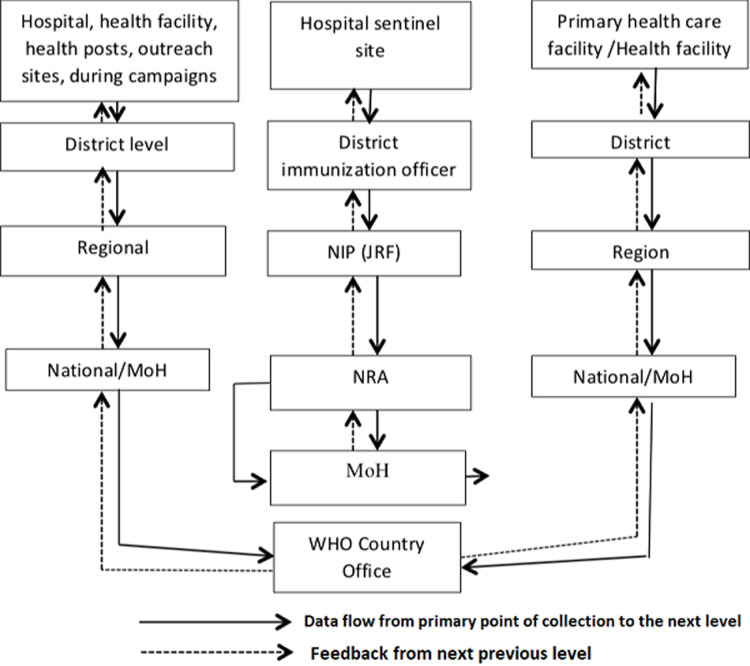
proposed organogram adapted for intussusception data flow chart

Figure 1 illustrates the proposed flow of AEFI data in countries. It outlines how cases are reported, data is transmitted from local to national levels and reporting to the global system. AEFI data is captured at a health facility/hospital by a health worker who fills in the AEFI investigation form and reports to the district immunization officer. This is then transmitted simultaneously to the Regional / Provincial Office and EPI Secretariat by fax or email. The Secretariat informs the NRA and PVC which are agents of MOH. The EPI/MOH will enter IS data into the JRF form and then communicate to WHO country office. This flow of data can occur within a short period of time. PVC will investigate and determine the causality of the vaccine if any. The investigation and assessment can take up to a week. Suspected cases of IS can be reported at various levels but assessed at the hospital by experienced paediatrician/surgeon trained on Brighton case definition and once confirmed by the experts, the case can be reported through the MOH structure below (Figure 1).

### Implementation

Further, we propose an initial pilot in selected 3-5 countries. This will provide an opportunity to assess the implementation and to gather more data on how to expand to all the countries in the region. Resources and technical support will be provided to these pilot countries to ensure that they have the minimum requirements for monitoring vaccine safety.

### Coordination of the implementation process

The Vaccine Preventable Disease (VPD) Programme of the University of North Carolina at Chapel Cluster and WHO Regional Office for Africa will coordinate the implementation of activities being proposed. This role will include organizing meetings, review of data and plans by countries. The VPD programme will also periodically convene meetings with countries to promote sharing of best practices.

### Monitoring and Evaluation (M&E)

The VPD programme of WHO/AFRO will also monitor the implementation, helping to identify any gaps and provide support to address them. The M&E will cover resources required, implementation of activities, outcomes including the reports of AEFIs in the Vigibase [11] as well as in the JRF.

### Perspective

As of July 2020, over 38 countries in the WHO/AFRO had introduced RRV in their NIP aimed at preventing severe dehydrating diarrhoea in children < 5 years and this is great progress in vaccine introduction over a period of over 8 years [12]. RVVs are safe with a low risk potential of causing IS in a small proportion of recipients of these vaccines. In sub-Saharan Africa, first severe episode of rotavirus diarrhoea occur early in life (2-5 months of age) [13]. The vaccines are given at 6 and 10 weeks and 6, 10 and 14 weeks of age for Rotarix and RotaTeq respectively. Both of these vaccines have low risk of IS especially with the first dose [10, 13]. However, IS also occurs naturally and the peak age is 5-8 months [8, 14-16].

The African Intussusception Surveillance Network has contributed to strengthening IS surveillance in the African region over the last 8 years. The second phase of IS surveillance is to establish a sustainable mechanism for the integration of IS monitoring into the routine AEFI reporting system, riding on the platforms that already exists in most sub-Saharan countries. It is envisaged that the integration will be gradual and rolled out as more experience is gained by EPI and districts health workers. There will be need to train the health workers on how to identify and report the cases to surgeon or paediatrician in hospitals with appropriate expertise and infrastructure. IS presents as acute or subacute abdomen in young infants. Because of the classical presentation of quadruple clinical features of bloody diarrhea, vomiting, abdominal distention and pain, this is commonly diagnosed as dysentery in sub-Saharan Africa [8]. Coupled with late presentation and delayed diagnosis in health facilities these factors lead to poor outcomes. Mortality rates ranging from 5% to 20% have frequently been quoted but this is certainly improving as there is sensitization and training in large hospitals during the IS surveillance project [6-8]. However, the situation remains largely unknown in district hospitals where there is no expertise to make a diagnosis of IS as most patients would be treated for dysentery as per Integrated Management of Childhood Illnesses guidelines which are followed in most health facilities outside the large referral teaching hospitals which have expertise in paediatrics and paediatric surgery [8, 14].

It is hoped that IS surveillance will be rolled out to other provincial / regional hospitals which were not in the initial sentinel sites surveillance and eventually to district hospitals which will provide the much-needed expertise in paediatrics and surgery though this will be country specific. Integration of IS surveillance to monitor RVVs AEFI into the existing system will increase the awareness of health workers in sub-Saharan Africa across the whole spectrum of health facilities including the lowest. As all the health facilities will be reporting on IS, a young infant who presents with dysentery-like illness will be evaluated better as a diagnosis of IS will be entertained much earlier thereby improving the outcome. Integration of IS surveillance into the AEFI platform is feasible and will also improve surveillance of other EPI targeted diseases. Furthermore, there is a lot of good will by the cooperating partners in the health sector to support vaccine safety monitoring and the time is now.

### Conclusion

This commentary raises key issues for consideration during the planning and implementation of integration of IS as part of routine AEFI. The need for robust systems of pharmacovigilance in Low and Low Middle Income Countries (LMICs) is critical as new vaccines are being introduced in these regions of the world. These new vaccines are unique to the weak health systems in the African region where systems for monitoring AEFIs are also inadequately resourced. Transition of the IS system which has collected and analyzed high quality data in support of rotavirus vaccine introduction in the region, will demonstrate the capacity of countries to monitor the safety of vaccines. This will increase the sensitization of health workers even at the lowest level. Furthermore, IS integration initiative will invigorate and strengthen AFEI monitoring and existing reporting platform. The lessons learned will inform safety monitoring of other new vaccines including vaccines against COVID-19.

### Disclaimer

The views expressed in this commentary are solely those of authors and do not necessarily represent the official position of WHO or the University of Zambia, School of Medicine.
